# The Genomic Architecture of Hidradenitis Suppurativa—A Systematic Review

**DOI:** 10.3389/fgene.2022.861241

**Published:** 2022-03-23

**Authors:** Nikolai Paul Pace, Dillon Mintoff, Isabella Borg

**Affiliations:** ^1^ Center for Molecular Medicine and Biobanking, University of Malta, Msida, Malta; ^2^ Department of Pathology, Faculty of Medicine and Surgery, University of Malta, Msida, Malta; ^3^ Department of Dermatology, Mater Dei Hospital, Msida, Malta; ^4^ Department of Pathology, Mater Dei Hospital, Msida, Malta

**Keywords:** hidradenitis suppurativa, genetics, pathophysiology, gamma secretase (γ-secretase), familial

## Abstract

Hidradenitis suppurativa is a chronic, suppurative condition of the pilosebaceous unit manifesting as painful nodules, abscesses, and sinus tracts mostly in, but not limited to, intertriginous skin. Great strides have been made at elucidating the pathophysiology of hidradenitis suppurativa, which appears to be the product of hyperkeratinization and inflammation brought about by environmental factors and a genetic predisposition. The identification of familial hidradenitis suppurativa has sparked research aimed at identifying underlying pathogenic variants in patients who harbor them. The objective of this review is to provide a broad overview of the role of genetics in various aspects of hidradenitis suppurativa, specifically the pathophysiology, diagnosis, and clinical application.

## Introduction

Hidradenitis suppurativa (HS) is a chronic, suppurative disorder characterized by inflammation and hyperkeratinization at the pilosebaceous unit (PSU). The condition manifests as tender nodules, draining abscesses and sinuses mostly in (but not limited to) intertriginous skin (von Laffert et al., 2011; Danby et al., 2013). The latest estimate of overall HS prevalence is 0.4% (95%CI, 0.26–0.63%) (Jfri et al., 2021). HS can arise as an isolated condition, in a syndromic form or in the setting of other cutaneous conditions (for example Dowling-Degos Disease). The pathophysiology of HS is complex and is strongly determined by environmental and lifestyle factors such as smoking and obesity. These factors interact with specific physical triggers, namely friction, sweat, increased temperature, and changes in the cutaneous microbiome to drive disease risk (Mintoff et al., 2021a).

HS has an underlying genetic etiology. The link between HS and a genetic predisposition has long been suspected. In the 1980s Fitzsimmons and Guilbert published evidence in favor of autosomal dominant or familial HS (fHS) (Fitzsimmons et al., 1984; Fitzsimmons et al., 1985; Fitzsimmons and Guilbert, 1985); however the causative gene remained elusive. Initial studies performed by Gao *et al.* suggested a possible HS locus at 1p21.1-1q25.3 (Gao et al., 2006). Based on these findings, linkage analysis was performed on HS-patients with a strong family history however, the results obtained failed to identify the causative locus (Saleh Al-Ali et al., 2010). Following the advent of whole exome sequencing (WES), Gao *et al* later described the c.210_211delAG *NCSTN* variant which segregated with disease in multiple family members affected by HS (Liu et al., 2011). Since then, several other investigators have provided evidence supporting a monogenic etiology in fHS, mostly involving loci encoding proteins of the γ-secretase complex (GSC) (OMIM #142690, #613736, #613737). These initial discoveries have fueled a strong drive to explore the role of genetic susceptibility in HS with the aim of better characterizing the pathophysiology of the disease.

The mechanisms and extent to which the combination of genomic and non-genomic factors determine disease manifestation, its phenotype and response to therapy are not fully understood. The increasing availability and affordability of genomic sequencing in both clinical and research settings, as well as more robust methods of interrogating the human genome has resulted in a surge of genetic data on HS. This genetic data is supported by *in vitro* and *in vivo* functional studies in a minority of cases. Nevertheless, a comprehensive understanding of the role of rare *vs*. common genetic factors that drive HS is lacking.

This systematic review aims to compile, analyze and present the extant literature pertaining to the genetic architecture of HS. Specifically, we aim to 1) comprehensively describe genetic variants that have been associated with HS phenotypes 2) evaluate the impact of selected missense variants on protein structure and stability using *in-silico* tools 3) explore genotype-phenotype associations and 4) discuss HS genetics in the context of disease subtypes.

## Methods

### Literature Search

A systematic literature search pertaining to hidradenitis suppurativa and genetic variation was conducted in PubMed/MEDLINE, Science Direct and Google Scholar databases. A comprehensive search strategy was based on the following combinations of free text keywords and Medical Subject Heading (MeSH) terms: “hidradenitis”, “suppurativa”, “acne”, “inversa”, “genetic”, “familial”, “secretase”, “nicastrin”, “*NCSTN*”, “*PSENEN*”, “presenilin”, “*PSEN*”, “*APH*”, “*NOD2*”, “*PSTPIP1*”, “*MEFV*”, “Syndrome”, “PASH”, “PAPASH”, “Pyoderma”, “SAPHO”, “PASS”, “Dowling-Degos”, “Mediterranean”. The Boolean operators used were “AND” and “OR”. The search covered articles published between January 1980 and January 2022, and was restricted to articles published in the English, Italian and Spanish languages. Furthermore, handsearching and citation review of relevant studies was also conducted to identify studies that were not captured by the electronic database search. Published original studies investigating the genetics of HS in its isolated or syndromic forms through both targeted and untargeted genomic approaches were eligible for inclusion. We excluded studies with 1) absent genetic data 2) duplicate data pertaining to the same proband in separate publications 3) conference proceedings, reviews, editorial letters or comments and 4) studies not directly investigating the association of specific genes with HS phenotypes.

### Data Extraction

Articles identified from the literature search were screened for duplicates. All studies deemed to be potentially eligible for inclusion were reviewed and data extracted by the investigators. Any discrepancies were resolved by consensus.

The following information was extracted from each eligible article 1) primary author and year of publication 2) locus, the specific genetic variant identified and segregation data when available 3) age at presentation of first lesion 4) primary anatomic sites affected and 5) data on comorbid risk factors, specifically obesity and smoking status.

### Data Analysis

Identified variants were categorized as missense, nonsense, frameshift indels, splice-site and non-coding regions according to their effect on translation. VarSeq software (Golden Helix) was used for variant interpretation and annotation (Kopanos et al., 2019). Variant pathogenicity was classified according to guidelines from the American College of Medical Genetics/Association for Molecular Pathology (ACMG/AMP) (Richards et al., 2015). These guidelines standardize variant classification by stratification into five categories (pathogenic, likely pathogenic, uncertain significance, likely benign, benign) based on a combination of computational, population, functional and segregation data. Rare variants were considered to be of uncertain significance (VUS) if there is limited or contradictory clinical or functional evidence for pathogenicity. The Human Gene Mutation Database, (HGMD), dbSNP and ClinVar databases (ncbi.nlm.nih.gov/clinvar/) were also interrogated to identify any respective studies and ontologies. To explore genotype-phenotype associations, variants in the GSC genes were considered as an aggregate category and contrasted against variants in genes not forming part of this complex.

### Protein Structure Analysis and Molecular Modelling

The impact of missense variants on protein structure was evaluated through molecular modelling. The DynaMut webserver was used to predict the effects of amino acid substitutions on protein stability, flexibility and to analyze interactions amongst amino acid residues (Rodrigues et al., 2018). The predicted change in stability between the wild type and variant structures derived from SDM, DUET, and mCSM algorithms is reported as ΔΔG in kcal/mol, with negative values indicative of destabilizing variants. Dynamut also reports vibrational entropy changes (ΔΔS_Vib_ in kcal mol^−1^K^−1^) calculated by ENCoM using normal-mode analysis to depict the substitution’s effect on structure flexibility or rigidity. To further categorize the structural impact of missense variants, the Missense3D and HOPE webservers were used. Missense3D assesses 17 different structural features that are essential for protein conformation and stability, such as stearic clashes and disallowed phi/psi angles (Venselaar et al., 2010; Ittisoponpisan et al., 2019). A comprehensive graphical summary of identified GSC variants was generated using ProteinPaint (Zhou C et al., 2016).

### Statistics

The characteristics of the cohort are summarized using descriptive statistics. Normality of age at first lesion was assessed by the Shapiro–Wilk and Kolmogorov-Smirnoff tests. This exhibited a skewed non-normal distribution; non-parametric statistics with medians and interquartile ranges are presented. To compare differences in quantitative variables between two categories, the independent-samples Mann–Whitney *U* test was applied. The chi-square test was used to compare categorical variables. A *p*-value of <0.05 was considered statistically significant.

## Results

**FIGURE 1:**
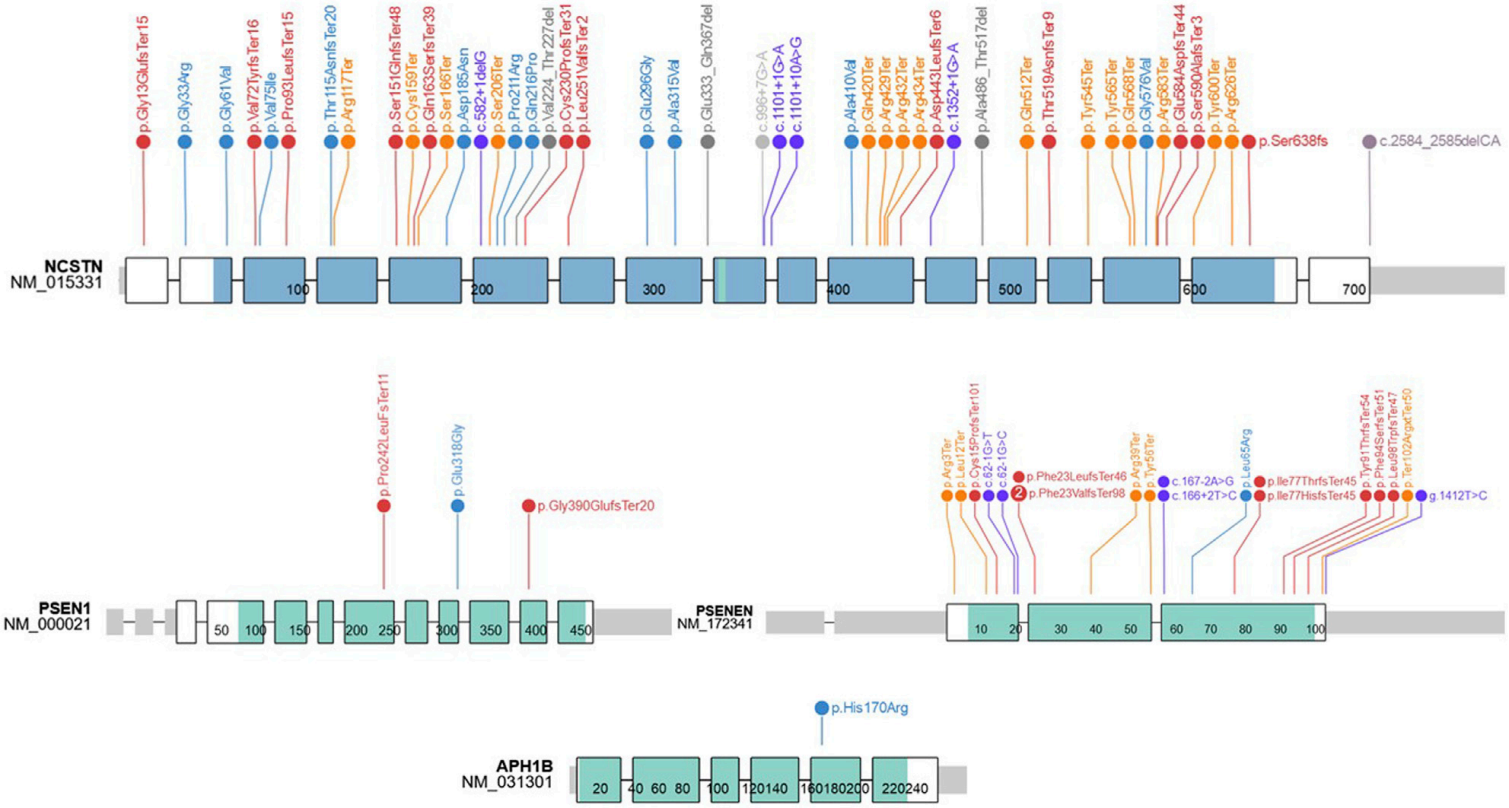
HS-associated variants in genes encoding proteins of the gamma secretase complex.

In total, the literature search identified 88 published variants implicated in HS. Approximately 83% of published variants involve the four genes encoding protein subunits of the multimeric GSC (*NCSTN* 54.5%, *PSENEN* 24%, *PSEN* 3.4% and *APH* 1%) (Figure 1). The remaining variants localized to *PSTPIP1, MEFV, NLRP3, IL1RN, NOD2* and *POFUT* loci, which have been variably associated with non-syndromic forms of HS. A summary of the identified variants, their pathogenicity classifiers and phenotypes are provided in Table 1. The reported median age at first lesion was 17 years (min 10 years—max 67 years). The axillae and groin were the most frequently reported affected sites. Clear segregation with the HS phenotype was documented by 78% of reports. The identified variants were classified as pathogenic or likely pathogenic in 66% of cases, and as VUS or VUS leaning pathogenic in 18% of cases according to the ACMG-AMP consensus criteria. At the protein level, variants were classified as missense (29.5%), nonsense (24%), frameshift indels (28.4%), splice donor/acceptor site (15.9%) and in non-coding regions (2.3%). Lifestyle factors that associate with HS predisposition and outcome, such as obesity and smoking status were inconsistently reported in the literature.

**TABLE 1:** A summary of described genetic variants, their protein effect, ACMG classification and phenotypic associations. *SAPHO ^*▲*^ *PASH • PAPASH* ^*$*^ *PPHSF* ^***+***^ *HS-DDD*.

Gene	Exon/Intron	Variant	Protein	ACMG classification	Variant Segregation	Age of 1st Lesion in the Proband	Involved Skin	Obesity and smoking Status	Group
*NCSTN*	1	NM_015331.3:c.38delG	p.Gly13GlufsTer15	Likely pathogenic	Sporadic	10	Axilla, inguinal, gluteal	Not Specified	Vural et al. (2021)
*NCSTN*	2	NM_015331.3:c.97G > A	p.Gly33Arg	Uncertain significance with some pathogenic evidence	Yes	Not Specified	Axilla, neck, trunk, axilla, gluteal, extremity	Not specified	Takeichi et al. (2020)
*NCSTN*	2	NM_015331.3:c.182G > T	p.Gly61Val	Uncertain significance leaning pathogenic	Yes	17	Not Specified	Obese, Non-smoker	Duchatelet et al. (2020)
*NCSTN*	3	NM_015331.3:c.210_211delAG	p.Val72TyrfsTer16	Pathogenic	Yes	Not specified	Abdomen, back, gluteal	Not Specified	Liu et al. (2011)
*NCSTN*	3	NM_015331.3:c.218delC	p.Pro93LeufsTer15	Pathogenic	Yes	24	Face, Neck, Back, Gluteal, Groin	Not specified	Wu et al. (2018)
*NCSTN*	3	NM_015331.3:c.223G > A	p.Val75lle	Benign	Yes	16	Face, Neck, Mons pubis, Genitals	Not Specified	Zhang et al. (2013)
*NCSTN*	3	NM_015331.3:c.278del ^*^	p.Pro93LeufsTer15	Pathogenic	Sporadic	40	Neck, Back, Axilla, Gluteal	Not obese	Li et al. (2018)
*NCSTN*	4	NM_015331.3:c.344_351del ^*▲*^	p.Thr115AsnfsTer20	Pathogenic	Proband adopted	11	Not Specified	Not obese	Duchatelet et al. (2015)
*NCSTN*	4	NM_015331.3:c.349C > T	p.Arg117Ter	Pathogenic	Yes	Not Specified	Not Specified	Not specified	Wang et al. (2010)
*NCSTN*	5	NM_015331.3:c.450_459del	p.Ser151GlnfsTer48	Pathogenic	Yes	11	Axilla, back, gluteal	Not specified	Wu et al. (2020)
*NCSTN*	5	NM_015331.3:c.477C > A	p.Cys159Ter	Pathogenic	Yes	17	Neck, Axillae, Gluteal, Popliteal fossae	Not obese	Xiao et al. (2016)
*NCSTN*	5	NM_015331.3:c.487delC	p.Gln163SerfsTer39	Likely Pathogenic	Yes	Not Specified	Axillary, inguinal and perineal fold	Not obese	Miskinyte et al. (2012)
*NCSTN*	5	NM_015331.3:c.497C > A	p.Ser166Ter	Likely pathogenic	Yes	14	Axillae, buttocks, groin and neck	Not Specified	Ma et al. (2014)
*NCSTN*	5	NM_015331.3:c.553G > A	p.Asp185Asn	Uncertain Significance	Sporadic	13	Axillae, chest, groin, buttocks	Smoker, Obese	Pink et al. (2012)
*NCSTN*	*Exon5/intron5 Donor splice site*	NM_015331.3:c.582+1delG	*Not applicable*	Pathogenic	Yes	Not Specified	Neck and perineal region	Not Specified	Nomura et al. (2013)
*NCSTN*	6	NM_015331.3:c.617C > A	p.Ser206Ter	Pathogenic	Yes	17	Head, Face, Axilla, Groin, Buttocks	Not obese	Shi et al. (2018)
*NCSTN*	6	NM_015331.3:c.632C > G	p.Pro211Arg	Likely Pathogenic	Sporadic	38	Axilla, trunk, buttock, groin	Not obese	Li et al. (2011)
*NCSTN*	6	NM_015331.3:c.647A > C	p.Gln216Pro	Uncertain Significance leaning pathogenic	Yes	18	Neck, Axillae, buttocks, and groin	Not specified	Zhang et al. (2013)
*NCSTN*	6	NM_015331.3:c.671_682del	p.Val224_Thr227del	Likely pathogenic	Yes	13	Axilla, Breast, Antecubital fossae, Neck	Not obese, Smoker	Mintoff et al. (2021c)
*NCSTN*	6	NM_015331.3:c.686_687dup	p.Cys230ProfsTer31	Pathogenic	Yes	Not Specified	Not specified	Not specified	Ratnamala et al. (2016)
*NCSTN*	7	NM_015331.3:c.751_752del	p.Leu251ValfsTer2	Pathogenic	Yes	13	Axilla, groin, buttock, back, lower abdomen, jaw	Not Specified	Zhang et al. (2021)
*NCSTN*	8	NM_015331.3:c.887A > G	p.Glu296Gly	Benign	Yes	Not Specified	Not specified	Not specified	Xu et al. (2016)
*NCSTN*	8	NM_015331.3:c.944C > T	p.Ala315Val	Uncertain significance leaning pathogenic	Yes	17	Buttock, Groin, Face, Scalp, Axillae, Back	Not specified	Zhang S et al. (2016)
*NCSTN*	8	NM_015331.3:c.996+1G > A	p.Glu333_Gln367del	Pathogenic	Not Specified	Not specified	Not Specified	Not Specified	Pink et al. (2016)
*NCSTN*	*Intron 8*	NM_015331.3:c.996+7G > A	*Not Applicable*	Benign	Sporadic	35	Axillae, Groin, Buttocks	Smoker, Obese	Pink et al. (2012)
*NCSTN*	Exon 9/intron 9 donor splice site	NM_015331.3:c.1101+1G > A	*Not applicable*	Pathogenic	Yes	16	Axillae, suprapubic area, groin, buttocks, thighs and neck	Smoker, Obese	Pink et al. (2011)
*NCSTN*	*Donor Splice site of exon 9*	NM_015331.3:c.1101 + 10A > G	*Not Applicable*	Uncertain significance	Sporadic	16	Axillae, Groin, Buttocks, Genitalia	Smoker, Not obese	Pink et al. (2012)
*NCSTN*	11	NM_015331.3:c.1229C > T	p.Ala410Val	Benign	Sporadic	32	Severe	Non-smoker, Not obese	Liu et al. (2016)
*NCSTN*	11	NM_015331.3:c.1258C > T	p.Gln420Ter	Pathogenic	Yes	Not specified	Neck, Hip	Non-Obese	Jiao et al. (2013)
*NCSTN*	11	NM_015331.3:c.1285C > T	p.Arg429Ter	Pathogenic	Yes	15	Face, abdomen, limbs, Gluteal	Non-smoker	Nishimori et al. (2020)
*NCSTN*	11	NM_015331.3:c.1294C > T	p.Arg432Ter	Pathogenic	Not Specified	Not specified	Gluteal, Groin and Perineum	Not obese	Lin et al. (2021)
*NCSTN*	11	NM_015331.3:c.1300C > T	p.Arg434Ter	Pathogenic	Yes	Puberty	Axillary, inguinal and perineal fold	Non-Obese	Miskinyte et al. (2012)
*NCSTN*	11	NM_015331.3:c.1325_1326insGTTGTTCTGTAGTGGC	p.Asp443LeufsTer6	Pathogenic	Not Specified	Not Specified	Not Specified	Not Specified	Plenary Lectures, (2019)
*NCSTN*	*Intron 11 Splice donor site*	NM_015331.3:c.1352+1G > A	*Not applicable*	Pathogenic	Yes	Not Specified	Not Specified	Not Specified	Liu et al. (2011)
*NCSTN*	*Intron 13 Splice site*	NM_015331.3:c.1551+1G > A	p.Ala486_Thr517del	Pathogenic	Yes	Not Specified	Typical and Atypical areas	Not specified	Wang et al. (2010)
*NCSTN*	13	NM_015331.3:c.1534C > T	p.Gln512Ter	Pathogenic	Not specified	Not Specified	Not Specified	Not specified	He et al. (2019)
*NCSTN*	14	NM_015331.3:c.1555dupA	p.Thr519AsnfsTer9	Pathogenic	Not specified	19	Neck, axillae, back, upper chest, gluteal and buttocks	Not specified	Qian et al. (2021)
*NCSTN*	14	NM_015331.3:c.1635C > G ^▲^	p.Tyr545Ter	Pathogenic	Yes	10	Axillae, Chest	Not specified	Faraji Zonooz et al. (2016)
*NCSTN*	15	NM_015331.3:c.1695T > G	p.Tyr565Ter	Likely pathogenic	Yes	26	Neck, axillae, gluteal and groins	Not obese	Li et al. (2011)
*NCSTN*	15	NM_015331.3:c.1702C > T	p.Gln568Ter	Pathogenic	Yes	15	Face, Neck, Buttocks	Not specified	Nomura et al. (2014)
*NCSTN*	15	NM_015331.3:c.1727G > T	p.Gly576Val	Uncertain significance leaning pathogenic	Familial but not studied	23	Not specified	Not obese, Non smoker	Duchatelet et al. (2020)
*NCSTN*	15	NM_015331.3:c.1747C > T	p.Arg583Ter	Pathogenic	Yes	25	Inguinal, genital	Non obese, Non-smoker	Garcovich et al. (2020)
*NCSTN*	15	NM_015331.3:c.1752delG	p.Glu584AspfsTer44	Pathogenic	Yes	Not specified	Typical and atypical areas	Not specified	Wang et al. (2010)
*NCSTN*	15	NM_015331.3:c.1768A > G	p.Ser590AlafsTer3	Likely benign	Yes	Not specified	Axillary, inguinal and perineal fold	Non-obese	Miskinyte et al. (2012)
*NCSTN*	16	NM_015331.3:c.1800_1801delTG	p.Tyr600Ter	Likely pathogenic	Yes	Not Specified	Not specified	Not specified	Ratnamala et al. (2016)
*NCSTN*	16	NM_015331.3:c.1876C > T	p.Arg626Ter	Pathogenic	Not Specified	Not Specified	Not Specified	Not Specified	Plenary Lectures, (2019)
*NCSTN*	16	NM_015331.3:c.1912_1915delCAGT	p.Ser638fs	Pathogenic	Yes	Before 20	Axillary, Inguinal, Back, nape and auricular region	Ex-Smoker, non-obese	Vossen et al. (2020b)
*NCSTN*	3′UTR	NM_015331.3:c.2584_2585delCA	Reduced expression	Pathogenic*	Sporadic	Not specified	Gluteal	Not Specified	Xiao et al. (2018)
*PSEN1*	7	NM_000021.4:c.725delC	p.Pro242LeufsTer11	Pathogenic	Yes	Not specified	Axilla, groin and Gluteal	Not specified	Wang et al. (2010)
*PSEN1*	9	NM_000021.4:c.953A > G	p.Glu318Gly	Likely benign	Not specified	Not specified	Not specified	Not specified	Ingram et al. (2013)
*PSEN1*	11	NM_000021.4:c.1167_1168insGA	p.Gly390GlufsTer20	Pathogenic	Yes	17	3	Obese, smoker	Duchatelet et al. (2020)
*PSENEN*	2	NM_172341.4:c.13C > T	p.Arg3Ter	Pathogenic	Yes	11	Not Specified	Not Specified	Qian et al. (2021)
*PSENEN*	2	NM_172341.4:c.35T > A^**+**^	p.Leu12Ter	Pathogenic	Yes	Not specified	Inguinal folds, Genitals	Obese, smoker	Ralser et al. (2017)
*PSENEN*	2	NM_172341.4:c.43_56del	p.Cys15ProfsTer101	Pathogenic	Yes	54	Axilla and buttocks	Not obese (according to pictures) smoking history not specified	Kan et al. (2018)
*PSENEN*	Splice Site	NM_172341.4:c.62-1G > C ^**+**^	*Not applicable*	Pathogenic	Yes	Not Specified	Axillae, inguinal folds, genitals	Obese, Smoker	Ralser et al. (2017)
*PSENEN*	Splice Site	NM_172341.4:c.62-1G > T ^**+**^	*Not applicable*	Pathogenic	Not specified	16	Axillae, Face and trunk	Not obese, Smoking history not specified	Peter et al. (2021)
*PSENEN*	Splice Site	NM_172341.4:c.66dup ^**+**^	p.Phe23ValfsTer98	Pathogenic	Sporadic	Not specified	Not specified	Not specified	Duchatelet et al. (2020)
*PSENEN*	3	NM_172341.4:c.66delG	p.Phe23LeufsTer46	Pathogenic	Yes	16	Nape, upper back and buttocks	Not obese according to clinical photos, Smoking history not specified	Xiao et al. (2020)
*PSENEN*	3	NM_172341.4:c.66_67insG	p.Phe23ValfsTer98	Pathogenic	Yes	15	Inframammary fold, axillae, groin	Not obese, Non-smoker	Pink et al. (2011)
*PSENEN*	3	NM_172341.4:c.115C > T ^**+**^	p.Arg39Ter	Pathogenic	Yes	Not Specified	Not Specified	Obese, Smoker	Ralser et al. (2017)
*PSENEN*	Intronic	NM_172341.4:c.166+2T > C ^**+**^	*Not applicable*	Pathogenic	Yes	13	Not Specified	Not obese, Non-smoker	Duchatelet et al. (2020)
*PSENEN*	Intronic	NM_172341.4:c.167–2A > G ^**+**^	*Not applicable*	Pathogenic	Yes	15	Face, neck, trunk	Not specified	Zhou X et al. (2016)
*PSENEN*	4	NM_172341.4:c.168T > G ^**+**^	p.Tyr56Ter	Pathogenic	Sporadic	Second decade	*“Flexural areas”*	Not Specified	Pavlovsky et al. (2018)
*PSENEN*	4	NM_172341.4:c.194T > G ^**+**^	p.Leu65Arg	Pathogenic	Yes	Not specified	Axilla, Neck, Perineum	Not Specified	Li et al. (2017), Zhou C et al. (2016)
*PSENEN*	4	NM_172341.4:c.228_229insCACC ^▲^	p.Ile77HisfsTer45	Pathogenic	Yes	22	Nape, Axilla, buttocks	Not specified	Zhang et al. (2020)
*PSENEN*	4	NM_172341.4:c.229_230insCACC	p.Ile77ThrfsTer45	Pathogenic	Not specified	Not Specified	Not Specified	Not specified	Zhou et al. (2021)
*PSENEN*	4	NM_172341.4:c.271delT	p.Tyr91ThrfsTer54	Pathogenic	No Incomplete penetrance	“Mean 15.15”	Not Specified	Not Specified	Theut Riis et al. (2020)
*PSENEN*	4	NM_172341.4:c.279delC	p.Phe94SerfsTer51	Pathogenic	Yes	Not specified	“*Typical and atypical”*	Not specified	Wang et al. (2010)
*PSENEN*		NM_172341.4:c.292del	p.Leu98TrpfsTer47	Pathogenic	No Incomplete penetrance	“MEAN15.5”	Not Specified	Not Specified	Theut Riis et al. (2020)
*PSENEN*	4	NM_172341.4:c.304T > A^**+**^	p.Ter102ArgxtTer50	Likely pathogenic	Not specified	18	Hurley 3	Not-obese, non-smoker	Duchatelet et al. (2020)
*PSENEN*	Splice Site	NM_172341.4:g.1412T > C ^**+**^	*Not applicable*	Pathogenic	Yes	Not Specified	Axillae, Inframammary region, inguinal folds	Non-smoker, obese	Ralser et al. (2017)
*APH1B*	5	NM_031301.4:c.509A > G	p.His170Arg	Uncertain Significance	Incomplete penetrance	Mean 15.5	Typical and atypical	Not specified	Theut Riis et al. (2020)
*PSTPIP1*	Promotor region	ENST00000558012.6:c.-413_-402dupCCTGCCTGCCTG ^**•**^	*Not applicable*	Benign	Not specified	15	Hurley Stage III	Not specified	Vural et al. (2019)
*PSTPIP1*	11	ENST00000558012.6:c.748G > C ^**•**^	p.Glu250Gln	Likely pathogenic	Sporadic		Not specified	Does not look obese on pictures, Smoking history not specified	Kotzerke et al. (2021)
*PSTPIP1*	11	ENST00000558012.6:c.764C > T^**$**^	p.Thr255Met	Benign	Not Specified	67	Buttocks, Hips	Not Specified	Hieta et al. (2021)
*PSTPIP1*	10	ENST00000558012.6:c.831G > T ^•^	p.Glu277Asp	Uncertain Significance leaning pathogenic	Not specified	14	Axilla	N/A	Marzano et al. (2013)
*PSTPIP1*	14	ENST00000558012.6:c.1034A > G ^▲^	p.Tyr345Cys	Uncertain significance	Yes	18	Axillae, Groin	Not Specified	Saito et al. (2018)
*PSTPIP1*	15	ENST00000558012.6:c.1213C > T^▲^	p.Arg405Cys	Conflicting interpretation of pathogenicity	Sporadic	27	Sacrococcygeal region, Intergluteal folds, perineal region	Non-smoker/Not obese	Calderón-Castrat et al. (2016)
*POFUT1*	4	NM_015352.2:c.430-1G > A *	*Consensus splice site mutation*	Pathogenic	Not Specified	23	Groin and Axillae	Not Specified	González-Villanueva et al. (2018)
*MEFV*	*10*	NM_000243.3:*c.2177T > C*	*p.Val726Ala*	*Pathogenic*	*Yes*	*18*	*Back, thighs, groin*	*Non-smoker Non-obese*	Jfri et al. (2020)
*NLRP3*	4	ENST00000336119.8:c.2107C > A ^▲^	p.Gln703Lys	Benign	Not specified	Not specified (Age at diagnosis given)	Face, trunk, lower extremities, axillary and inguinal folds, anogenital area	Not specified	Marzano et al. (2014)
*IL1RN*	4	NM_000577.5:c.370G > A^▲^	p.Ala106Thr	Benign	Not specified	Not specified (Age at diagnosis given)	Face, trunk, lower extremities, axillary and inguinal folds, anogenital area	Not specified	Marzano et al. (2014)
*NOD2*	8	NM_001370466.1:c.2023C > T ^▲^	p.Arg675Trp	Benign	Not specified	Not specified (Age at diagnosis given)	Face, trunk, lower extremities, axillary and inguinal folds, anogenital area	Not specified	Marzano et al. (2014)
*NOD2*	8	NM_022162.3:c.2722G > C ^▲^	p.Gly908Arg	Benign	Not specified	Not specified (Age at diagnosis given)	Face, trunk, lower extremities, axillary and inguinal folds, anogenital area	Not specified	Marzano et al. (2014)
*NOD2*	10	NM_022162.3 c.2923C > G^▲^	p.Leu975Val	Uncertain significance	Yes	19	Axilla, Inner thighs	Non-smoker, non-obese	Jfri et al. (2020)

No significant difference in the reported age at first lesion was identified between carriers of variants in GSC and other loci (17 vs. 19 years, *p* = 0.112). Similarly, no difference in age at first lesion between *NCSTN* variant carriers and non-*NCSTN* variant carriers was identified (17 vs. 18 years, *p* = 0.345). Carriers of pathogenic variants had a marginally lower age at first lesion than carriers of VUS or benign variants (16 vs. 18 years *p* = 0.048). A significantly higher proportion of pathogenic variants was located in GSC genes compared to other loci (74 *vs*. 26.7%, χ2 = 12.1, *p* = 0.001). As expected, variants that disrupt translation through nonsense, frameshift or splice-altering effects are more likely to be pathogenic than missense variants (91.4% *vs*. 8.6%, χ2 = 42.2, *p* < 0.01). No significant difference in obesity or smoking status was noted when probands carrying pathogenic variants were compared to those carrying non-pathogenic variants, although these parameters were inconstantly reported in the literature. No association between the primary anatomic sites involved by HS lesions and gene (*NCSTN* vs. non-*NCSTN*) or the ACMG pathogenicity classifier (pathogenic *vs*. non-pathogenic variants) was noted. Table 2 contrasts the salient clinical and genetic features between GSC variant carriers and non-carriers.

**TABLE 2:** A comparison of the salient clinical and genetic findings between HS cases bearing variants in γ-secretase complex genes and HS cases with variants at other loci. All non-GSC genes (*PSTPIP, MEFV, NOD2, IL1RN* and *NLRP3*) that have been associated with HS subtypes are considered as an aggregate category.

		GSC Genes (n = 73)	Non-GSC Genes (n = 15)
Age of first lesion (years)	Median (IQR)	17 (9)	19 (9)
Protein effect	Missense	17.8%	86.7%
	Nonsense	28.8%	0.0%
	Frameshift indels	34.2%	0.0%
	Splice site	17.8%	6.7%
	UTR/non-coding	1.4%	6.7%
	ACMG/AMP classification	Pathogenic	74.0%	26.7%
VUS		20.5%	20.0%
Benign		5.5%	53.3%
Segregation	Segregation	79.7%	60.0%
	Sporadic	15.3%	40.0%
	Incomplete Penetrance	5.1%	0.0%
% Obese		32.3%	0.0%
% Smokers		50.0%	0.0%

Abbreviations GSC - γ-secretase complex. IQR, interquartile range; VUS, variants of uncertain significance; UTR, untranslated region.

For selected missense variants we explored the structural impacts of the substitution and assessed the change in ΔΔG and Δ vibrational entropy energy predictions between the wild-type and mutant structures. An overview of the findings is presented in Supplementary Table S1. Only five of 23 (21.73%) missense variants were predicted to exert conformationally deleterious effects as they impact critical structural residues (*NCSTN* p. Gly61Val, p. Gln216Pro, p. Glu296Gly, p. Gly576Val and *APH* p. His170Arg). Four of these variants have VUS/VUS leaning pathogenic classifications while the fifth (*NCSTN* p. Glu296Gly) is classified as benign based on allele frequency cut-offs. However, most missense variants reported in the literature demonstrated destabilizing effects with changes in conformational flexibility. These findings suggest that missense variants in HS-associated loci may contribute to disease by modulating protein activity in a variety of ways.

## Discussion

This review provides a comprehensive summary of individual studies that investigate the genomic etiology of HS in its various forms; published variants to date are collated and appraised using the conservative ACMG-AMP criteria, genotype-phenotype associations assessed, and the structural impact of missense variants evaluated computationally. Our findings expound the locus and allelic heterogeneity underlying this inflammatory disorder and demonstrate a lack of robust correlation with clinical phenotypes. Herein we outline the physiological relevance of the HS-associated loci.

Despite the high heritability estimates of HS (77–80%) only a minority of HS patients demonstrate a strong monogenic etiology in the context of familial or syndromic HS (5%) (Kjaersgaard Andersen et al., 2021). Nevertheless, common forms of the disease demonstrate familial segregation. A family history of HS was documented in 41% in a cohort of 271 pediatric HS patients (Liy-Wong et al., 2021). The high heritability estimates reported by van Straalen *et al* suggest that sporadic forms of HS have a strong genetic element that contributes to their causality, despite the absence of fully penetrant variants causing multigenerational disease (Straalen et al., 2020). To date, the exact nature of the genetic variants driving common forms of HS remains unelucidated.

### Based on Existing Knowledge on the Genomics of Hidradenitis Suppurativa, the Disease can Be Categorized as

1. Sporadic HS: HS with no identified genetic variation to date
2. Familial HS: HS with a strong family history and established underlying monogenic etiology
3. Syndromic HS: HS in the setting of constellation of other clinical manifestation (PASH, PAPASH, SAPHO)
4. HS+: HS in the setting of Dowling-Degos Disease (HS-DDD) or Familial Mediterranean Fever (HS-FMF).

### Familial Hidradenitis Suppurativa: Variation in γ-Secretase Complex Protein-Coding Genes; *Knocked down a Notch*

Genetic variants in the GSC account for the majority of identified variants in fHS and sHS cases, but for only a minority of cases of sporadic HS. Cohort studies showed a low prevalence of GSC variants in sporadic forms of HS. The largest multicenter study in cases of predominantly Caucasian ethnicity identified pathogenic GSC variation in only 12 out of 188 (6.4%) patients with HS, of which 51% had fHS (Duchatelet et al., 2020). Smaller scale cohort studies in the United Kingdom revealed no GSC pathogenic variants in HS patients irrespective of family history of the disease (Ingram et al., 2013).

The human GSC is a multimeric, intramembrane-cleaving proteases composed of four subunit domains namely: Nicastrin (NCSTN), Presenilin Enhancer 2 (PEN2), Presenilin 1 (PSEN1) or PSEN2 and Anterior Pharynx Defective (APH) 1A or B. This gives rise to at least six different GSCs, which assemble fully in the endoplasmic reticulum and are transported to the cell membrane (Capell et al., 2005). The GSCs are dynamic and can exist in three conformational states; extended, intermediate and compact (Elad et al., 2015). Cryo-electron-microscopy single-particle analysis has elucidated the three-dimensional structure of human GCS, which was shown to have a horseshoe-shaped transmembrane domain (spanning 19 transmembrane segments) and a large extracellular domain (Lu et al., 2014).

The most widely reported variants in fHS lie in the nicastrin-coding gene *NCSTN* (OMIM #142690). Nicastrin is the largest subunit of the GSC (accounting for two-thirds of the molecular mass of the entire complex), and is postulated to be the substrate-recruiting protein of the GSC [specifically at the DYIGS and peptidase-like (DAP) region in the ectodomain (ECD)] (Shah et al., 2005). Nicastrin is essential for the assembly (particularly of the transmembrane domain) (Shah et al., 2005), maturation and stability of the GSC (Elad et al., 2015). The protein has a bilobed ECD and a single transmembrane domain (TMD) at its C-terminus (Xie et al., 2014). The interface between the two lobes is maintained by extensive van der Waals contacts, amongst which those formed between Phe287 (of the large loop) nestled within a pocket of hydrophobic amino acids Phe103, Leu171, Phe176 and Ile180 (of the small loop), is highly conserved (Xie et al., 2014). A loop extending from the core of the small lobe of the nicastrin ECD forms a lid which covers the putative substrate binding site within the large lobe, amongst which the residue Trp164 is vital (Xie et al., 2014). This amino acid, as well as Pro-141, Trp-164, Asn-165 and Gly-168 have been shown to be highly conserved in the NCST “lid” domain, but not essential for regulating GSC activity, including Notch (Zhang X et al., 2016). Fluorescence imaging microscopy of intact cells detected conformational change of the nicastrin ECD, which is brought closer to the membrane core upon binding an inhibitor (Elad et al., 2015). Residues Tyr337 and Glu333 (Shah et al., 2005) are both located within the substrate biding pocket ECD (Xie et al., 2014). The importance of Glu333 in proteolysis has been confirmed by mutagenesis studies, demonstrating abolished GSC cleavage, activity and maturation after substituting the residue (Shah et al., 2005; Dries et al., 2009), thus dispelling assertions that Glu333 is only involved in GSC maturation (Chávez-Gutiérrez et al., 2008). Nicastrin also forms complexes with PSEN1 and PSEN2, establishing a “secretasome” which allows for intramembranous proteolysis of the transmembrane proteins, including Notch (Yu et al., 2000).

The Notch signaling pathway is a highly conserved pathway involved in cell-cell communication. It regulates cellular differentiation and proliferation in continually renewing adult tissues such as skin. In these tissues, the notch receptor is activated by various ligands and cleaved by the GSC, releasing its intracellular domain which translocates to the nucleus to regulate gene expression (Mumm and Kopan, 2000). Because of the important roles played by Notch in epidermal and follicular homeostasis as well as inflammation, Notch dysregulation has been touted to underpin the molecular basis of HS in patients with pathogenic variants in GSC-protein coding genes (Okuyama et al., 2008; Melnik and Plewig, 2013). Interaction of the Notch receptor with its ligand (delta or jagged) results in two subsequent proteolytic cleavages of the receptor, the first of which is catalyzed by ADAM-family metalloproteinases and the second by the GSC. The product of the second cleavage, Notch intracellular domain (NICD), subsequently translocates to the nucleus where it acts as a transcriptional regulator for various genes (Borggrefe and Oswald, 2009) after complexing with CBF1-Suppressor of Hairless-LAG1 (CSL) and the co-activator mastermind (Wilson and Kovall, 2006).

In human models, downregulation of Notch signaling pathway has been shown to perturb keratinocyte differentiation and result in uncontrolled proliferation, disorganization of the suprabasal layers of the epidermis as well as dermal invasion (Thélu et al., 2002). These findings were also replicated in murine models wherein alteration in notch signaling resulted in altered sebaceous gland differentiation and terminal differentiation of the epidermis (Pan et al., 2004; Blanpain et al., 2006; Wang et al., 2008). Loss of ADAMS10 (responsible for notch receptor cleavage) (Groot and Vooijs, 2012) in murine models has been shown to result in impaired epidermal differentiation resulting in various pathologies including epidermal hyperproliferation and cyst formation as a result of altered notch signaling (Weber et al., 2011). Studies have also demonstrated that ADAMS10 is downregulated in HS (Frew and Navrazhina, 2020). Dysregulation of ADAM10-Notch signaling axis has been shown to impair the epithelial barrier and favor cutaneous dysbiosis (favoring *Corynebacterium spp*) (Sakamoto et al., 2021). Potentially, the altered microbiome promotes chronic inflammation by triggering the innate lymphoid cell population in an IL-17R dependent manner (Sakamoto et al., 2021). These findings may partly underpin the dysregulated cutaneous microbiome that accompanies HS (Mintoff et al., 2021a). Murine models have also demonstrated that disruption of notch nuclear target *RBP-J* results in cyst formation and epidermal hyperkeratinization (Yamamoto et al., 2003). A study scrutinizing publicly available genomic data revealed significant downregulation of Notch 1–4, and suggests ADAM17 as a key mediator in the pathogenesis of HS (Frew and Navrazhina, 2020).

Evidence for the role of Notch in HS can also be drawn from HS + disease such as HS-DDD. DDD is an autosomal dominant genodermatosis characterized by flexural and reticulated pigmentation. The condition is attributed to heterozygous variants in *KRT5* (DDD1, OMIM #179850) at 12q13 and *POFUT1* (DDD2, OMIM #615327) at 20q11. (Crovato et al., 1983; Stephan et al., 2021). Dubbed as “clinical collision” diseases, HS-DDD provides avenues for understanding pathophysiology and phenotypes (McGrath, 2018). Both *POGLUT1* and *POFUT1,* causative genes in DDD, are established regulators of Notch pathway activity through their respective protein products namely protein-O glucosyltransferase 1 and protein O-fucosyltransferase 1 (Li et al., 2013; Basmanav et al., 2014). Congruently, patients with HS-DDD having underling *POGLUT1* pathogenic variants demonstrate abnormal expression of genes encoding the critical element of the Notch pathway (Pavlovsky et al., 2018). Further molecular evidence is derived from two patients with HS-DDD, where reduced expression of *NOTCH1* and *NCSTN* mRNA was demonstrated in lesional skin when compared to non-lesional skin, (Penha et al., 2020). HS-DDD with underlying *POFUT1* pathogenic variants has been suggested to share similar Notch disturbance; however robust serological and *in-vitro* studies are lacking (González-Villanueva et al., 2018). Indirect evidence supporting the role of Notch downregulation in HS is provided by a case report in which a patient treated with a notch signaling inhibitor AL101 (BMS-906024) developed HS (Wiggins and Chon, 2020).

Conflicting findings have also been reported. Nicastrin, Notch 1–3, PIK3R3 and AKT3 levels were found to be significantly higher in lesional skin of 60 HS patients when compared to healthy controls. In lesional skin, these proteins were significantly higher in patients with mild (Hurley stage 1) disease compared to those with moderate and severe disease (Hurley stage II and III), despite excluding smoking and obesity as confounding factors (Hessam et al., 2021). Nicastrin overexpression (particularly dermal) has been associated with hypertrophic scarring as well as with inhibition of Notch signaling resulting in the suppressed production of fibrotic factors such as collagen 1 and 3 and TGF-β1 (Chen et al., 2021). Functional studies have shown that various HS-inducing nicastrin missense variants are active and sustain Notch signaling, and therefore do not fully support the concept of notch as being the singular pathophysiological processes involved in NCSTN-associated HS (Zhang and Sisodia, 2015). Indirect evidence for upregulation of notch can be drawn from other inflammatory pathologies. In patients with psoriasis, the proinflammatory cytokine serum amyloid A (SAA), known to be highly elevated in sera of HS patients (Witte-Händel et al., 2019), has been shown to upregulate Notch1 activity (Rooney et al., 2014).

PSEN has also been implicated in Notch signaling. The homologs PSEN1/PSEN2 are the catalytic subunit of the GSC. PSEN1/GSC is widely distributed in the cell (including its plasma membrane) whilst PSEN2/GSC is mostly restricted to late endosomes and lysosomes (Sannerud et al., 2016). Auto-compensatory mechanisms maintain PSEN levels in equilibrium (Stanga et al., 2018). In animal models, only zebrafish affected by the familial acne inversa-like indel mutation *psen1. p*trp233fs (equivalent to human *PSEN1* codon P242) had shown a significant alteration in Notch signaling (as opposed to early onset familial Alzheimer disease mutants). Upregulation of genes involved in inflammation was also observed in these mutants (Barthelson et al., 2021). The apparent upregulation of Notch signaling may possibly be accounted for by transcriptional adaptation, the process by which fragments of mutated mRNA translocate to the nucleus leading to transcriptional modulation of “adapting genes” (Sztal and Stainier, 2020). HS patients with underlying *PSEN* variants are designated OMIM # 613737.

Variants of Presenilin enhancer 2 (*PSENEN*), which codes for presenilin enhancer 2 (PEN2) have also been described in both sporadic HS as well as HS + DDD. The role of PEN2 in GSC function and Notch signaling was found to be conserved across species (Francis et al., 2002). PEN2 is the last unit to be incorporated within the GSC, with a PEN2 “retention mechanism” ensuring that only fully assembled GSCs are released from the endoplasmic reticulum to the plasma membrane (Capell et al., 2005; Christoph et al., 2007). The incomplete penetrance of *PSENEN* pathogenic variants has been described in three families by Riis *et al* (Theut Riis et al., 2020). Potentially, this can be attributed to the fact that PEN2 is not part of the GSC proteolytic domain. Additionally, it is possible that monoallelic pathogenic variants may not cause disease in the absence of other risk factors. *In silico* studies further support the concept that heterozygous pathogenic variants in *NCSTN* and *PSENEN* are not sufficient to cause disease (Nomura et al., 2014; Theut Riis et al., 2020).

APH-1 is the least well-characterized locus in the setting of HS, with a single *APH-1B* variant (p.His170Arg) having been associated with HS (Theut Riis et al., 2020). The authors postulate that this variant is unlikely to be causative of HS, citing studies which demonstrate much higher APH-1A expression in skin and fibroblast models in which APH-1A exclusively is involved in Notch signaling (Theut Riis et al., 2020). APH1 is a 7-transmembrane helix protein expressed as two homologous isoforms in humans, encoded by two genes (APH1a on chromosome 1; APH1b on chromosome 15). Both APH-1A and APH-1B adopt a water channel topology and transport water across the plasma membrane (Aguayo-Ortiz and Dominguez, 2019). The conserved His170 residue together with His196 play a major role in water transportation across the lipid bilayer (Aguayo-Ortiz and Dominguez, 2019; Dehury and Kepp, 2021).

The significance of GSC variants and the disruption in Notch signaling pathways and its targets in HS, is still evolving. Further functional and multiomic studies are required to determine the definitive role of the Notch signaling pathway in skin disease, including HS (Brandão et al., 2021). A working model of HS pathophysiology suggests that underlying genetic variants lead to lower protein expression which becomes functionally relevant under cellular stress mediated by friction (obesity), temperature and dysbiosis (Pink et al., 2016).

### Syndromic Hidradenitis Suppurativa

In a subset of patients, HS develops as part of a constellation of other inflammatory, conditions (sHS). The classical HS syndromes in which genetic variation has been described include PASH (pyoderma gangrenosum, acne and suppurative hidradenitis), PAPASH (pyogenic arthritis + PASH) and SAPHO (synovitis, acne, pustulosis, hyperostosis and osteitis) syndromes (Garcovich et al., 2021a). A *PSTPIP1* pathogenic variant was recently identified in a patient with proctitis, pyoderma gangrenosum, HS and fever (dubbed “PPHSF” syndrome) (Hieta et al., 2021). Supplementary Table S2 summarizes the genetic variants associated with sHS.

Braun-Falco *et al.*, had documented the first two families with PASH. No pathogenic variants in *PSTPIP1* were detected; however afflicted patients had hemi-allelic increase of the CCTG microsatellite motif (Braun-Falco et al., 2012). Other cases of PASH without *PSTPIP1* pathogenic variants have also been reported (Gracia-Cazaña et al., 2015; Niv et al., 2017; Lamiaux et al., 2018). Similarly, no pathogenic variants were described in other forms of sHS namely, PsAPASH (Psoriatic arthritis and PASH) syndrome (Saraceno et al., 2015), PsAPSASH (Psoriasis, arthritis, pyoderma gangrenosum, synovitis, acne, suppurative hidradenitis) (Nikolakis et al., 2021) and PASS (pyoderma gangrenosum, acne, hidradenitis suppurativa and ankylosing spondylitis) (Bruzzese, 2012; Leuenberger et al., 2016) syndromes ***.*** Follicular occlusion syndromes such as the follicular occlusion triad [HS, acne conglobata and dissecting cellulitis of the scalp (Perifolliculitis capitis abscedens *et* suffodiens)] (Chicarilli, 1987) and the follicular tetrad (Vasanth and Chandrashekar, 2014) (follicular triad and pilonidal sinus) are described but likewise, they also lack an identified genetic driver.

*PSTPIP1* is the locus most frequently implicated in sHS that features in PASH, PAPASH and PPHSF syndromes. Interestingly, *PSTPIP1* variants have not been described in isolated forms of HS. *PSTPIP1* codes for Proline-serine-threonine phosphatase-interacting protein 1. It has been demonstrated that PSTPIP1 regulates the transition of macrophage’s podosomes to filopodia-like protrusions and modulates their invasive migration (Starnes et al., 2014). Cellular studies have also demonstrated that pyrin modulates the intracellular distribution of PSTPIP1, and co-localizes at the leading edge of cells mitigating cell migration (Shoham et al., 2003; Waite et al., 2009; Akkaya-Ulum et al., 2015). In fact, *PTSPIP1* mutant T-cells were shown to have altered f-actin polymerization (Janssen et al., 2018).

*MEFV* variants have been shown to be more frequent in patients with HS than healthy controls (Vural et al., 2019). A patient of Turkish origin suffering from comorbid PASH and familial Mediterranean fever (FMF) has been described. Targeted analysis in the *MEFV* gene identified the two heterozygous pathogenic variants (p.M680I and p. V726A); assessment of the rest of his genome was therefore lacking (Vural et al., 2017). Interestingly, a patient with PAPASH was heterozygous for a microsatellite elongation in the *PSTPIP1* promotor region, and homozygous for the pathogenic *MEFV* p. Met694Val variant. However, he was asymptomatic for FMF. The authors suggest that this could possibly be the result of aberrant mutant Pyrin-PTSPIP1 interaction (Vural et al., 2019). The cause-effect relationship underlying the coexistence of a systemic proinflammatory susceptibility typical of FMF with inflammation of the PSU remains to be established.

Cytokine IL-1β may be the common denominator linking variants described in sHS or HS in combination with pyoderma gangrenosum (Galimberti et al., 2016; Witte-Händel et al., 2019). The protein products encoded by *PSTPIP1* and *MEFV* interact in a multimeric protein complex that regulates assembly and activation of the inflammasome by promoting ASC oligomerization and Caspase-1 activation (Yu et al., 2007). The inflammasome regulates innate immunity and epithelial barrier defenses. On the other hand, gain of function mutations in *NLRP3* result in increased IL-1 [a defining feature of Cryopyrin-associated periodic syndrome (CAPS)] (Kuemmerle-Deschner et al., 2017). Conflicting results have been observed with regards to IL-1β levels in patients with sHS. No statistically significant differences in serum Il-1β levels were described between patients with PASH syndrome and healthy controls (Marzano et al., 2014). Conversely, PASS has been characterized as an IL-1-driven autoinflammatory disease which responds to treatment with the Il-1 Receptor antagonist anakinra (Leuenberger et al., 2016). Anakinra has been proven to be ineffective in the treatment of a young female with PASH syndrome (Staub et al., 2015).

HS has also been documented to manifest in the setting of specific chromosomal disorders, where it does not constitute a classical diagnostic feature. It has been speculated that diminished Notch receptor processing and signaling could account for HS is the setting of Trisomy 21; however a definitive functional correlation remains elusive (Gasparic et al., 2017). A case report also describes the occurrence of unilaterally distributed HS, possibly due to constitutional mosaicism in a patient with trisomy 1q (Skroza et al., 2019).

### Hidradenitis Suppurativa +

HS has been independently described in the setting of two other heritable conditions namely, HS-DDD and Familial Mediterranean Fever (HS-FMF).

A distinctive subtype of Dowling-Degos with HS (HS-DDD) is defined by heterozygous variants in *PSENEN* (OMIM # 613736) on 19q13. Patients having underlying *PSENEN* variants but suffering from DDD exclusively have been described (Ralser et al., 2017; Ren and Zeng, 2020). Interestingly, only obese family members harboring the pathogenic *PSENEN* c.62-1G > C splice variant manifested HS + DDD, whilst their lean, non-smoking relative who also harbored the same mutation manifested DDD exclusively (Ralser et al., 2017). Non-smoking, lean patients from another family having *PSENEN* 84_85insT variant manifested DDD without HS (Ralser et al., 2017). On the other hand, the c.216delC *PSENEN* variant was described in non-smoking, lean patients from two separate families manifesting DDD exclusively but also in an unrelated patient with DDD-HS, whose smoking history and weight were not documented (Ralser et al., 2017; Ren and Zeng, 2020; Theut Riis et al., 2020). This suggests that in the context of HS, *PSENEN* pathogenic variants exhibit incomplete penetrance and variable expressivity, and possibly only manifest disease in the setting of specific triggers such as obesity. A pathogenic *NCSTN* nonsense variant p. Arg583Ter (c.1747C > T) has also been described in a lean, non-smoking patient with HS-DDD. The variant segregated with the DDD phenotype, but not HS, in the proband’s daughter (Garcovich et al., 2020; Garcovich et al., 2021b). However, the pathogenicity of this variant and its relevance to HS-DDD has been disputed, mainly because the significance of co-existing *KRT5* variants was downplayed and deemed benign (Hermasch et al., 2020).

HS can occur in combination with other inherited autoinflammatory syndromes. Two patients with co-morbid HS - mevalonate kinase deficiency (Benhadou et al., 2021), an autosomal recessive inborn error of metabolism which leads to chronic inflammation, have been described. Various studies have also described the co-existance of an HS phenotype in patients bearing pathogenic variation at the *MEFV* locus (Abbara et al., 2017; Vural et al., 2017; Jfri et al., 2019; Vural et al., 2019). This gene encodes pyrin, a protein which modulates the activity of the GSC. These studies suggest that HS and FMF are autoinflammatory disorders that may possibly share converging pathophysiologic processes. Despite the systemic proinflammatory state in these conditions, proof of causal associations remains lacking.

### Genotype-Phenotype Correlations

Patients with HS exhibit extensive phenotypic heterogeneity making genotype-phenotype correlations difficult to establish (Kent, 2009). The task is further complicated by poor interrater reliability of HS phenotypes (van Straalen et al., 2018), differences in severity scoring (Zouboulis et al., 2019) as well as evidence of pleiotropism (Gratton et al., 2020). Notwithstanding, some general patterns are emerging, and encouraging results are emerging even from relatively low-powered studies (Marzano et al., 2022). HS patients having underlying *NCSTN* variants appear to have a follicular-type HS, in which comedones, papules and folliculitis predominate. Patients with this subtype of HS are likelier to manifest lesions in the nape and back and have co-morbid pilonidal sinus disease (PND) (Xu et al., 2016), a common co-morbidity and a possible intergluteal localization of disease in HS patients (Benhadou et al., 2019). Similarly, phenotyping of a large cohort of HS patients showed that cases exhibiting a follicular (LC2) type phenotype, typified by epidermal cysts, PND and comedones were more likely to have a family history of HS when compared to the patients with axillary-mammary (LC1) and gluteal (LC3) phenotypes (Canoui-Poitrine et al., 2013). An inverse correlation between LC1 HS phenotypes and *NCSTN* variants has been described (Frew et al., 2019). Patients with an endotype defined by GSC pathogenic variants and higher levels of serum IL-10 are more likely to be non-obese males with predominantly nodular lesions on the trunk and posterior sites and have a history of PND (referred to as “Cluster 1” HS patients). On the other hand, patients with high serum IL-1, IL-17, IL-16 and CRP are more likely to be obese and have later-onset disease, with tunnels and abscesses predominating (Cluster 2) (González-Manso et al., 2020). With regards to patients with sHS, both promoter and *PSTPIP1* variants were found to be significantly associated with syndromic forms of disease (Frew et al., 2019).

In a broader sense, HS patients having an affected first-degree relative develop more severe disease at a younger age than their parents. The gender of the affected parents also influences the resulting phenotype in the offspring, with patients having an affected mother more frequently reporting axillary involvement, and patients with affected fathers being significantly more likely to involve the buttocks and the genitals. The mean number of affected body sites was found to be significantly higher in patients whose father has HS then those with a maternal history of the condition (Plenary Lectures, 2019).

Patients who are obese and smokers and without any underlying genetic variants are more likely to present with a “wet” phenotype characterized by draining abscesses in intertriginous regions. Conversely, lean non-smokers with a known family history are more likely to develop a “dry” phenotype characterized by follicular lesions in atypical regions such as the nuchal area and antecubital fossae (Vossen et al., 2020a; Mintoff et al., 2021b), or with syndromic HS.

### Hidradenitis Suppurativa Genomics—Caveats and Challenges

The interpretation of genetic findings in HS presents several limitations. When compared to other common complex traits, there is a deficiency of large-scale genomic studies on ethnically diverse cohorts. The relative contribution of common *vs*. rare polymorphisms remains unascertained. Applying high-throughput sequencing studies to kindreds with early-onset familial or atypical HS phenotypes is a valid approach. However, variant prioritization and pathogenicity scoring can be complicated by pitfalls such as the overreliance on *in-silico* predictors and the use of inappropriate allele frequency cut-offs. Additionally, limited conclusions about the role of monoallelic variants causing recessive disorders can be drawn. It must be acknowledged that the assessment of allele frequencies in aggregate datasets unselected for disease, such as GnomAD, is a valuable approach. However, the presence of rare variants causative of late-onset disease in genomic databases can confound variant classification (Lek et al., 2016). Attributing causality to variants remains a considerable challenge, particularly for missense substitutions that are not structurally deleterious. Functional evaluation using *in-vitro* or *in-vivo* models are required to support pathogenicity and robustly define gene-disease associations for disputed loci. This is reinforced by conflicting interpretations of pathogenicity attributed to some variants in clinical databases. Furthermore, studies based on exome capture and sequencing may fail to identify deep intronic variants that modulate splicing or pathogenic structural variation. In the broader context of HS genomic architecture, it is essential to consider that studies sequencing cases with multigenerational early-onset disease are likely to skew towards the identification of high-penetrance variants. These represent the ‘low-hanging fruit’ of genomic discovery, at the expense of variants that lack adequate penetrance to drive familial segregation of disease. Plausibly, such intermediate penetrance variants predispose to later onset or milder disease.

This review is intrinsically limited by study selection criteria, and it is possible that some studies may not have been included (language other than English, Italian and Spanish, articles missing key data and conference proceedings). In addition, key phenotypic data, such as obesity and smoking status, as well as familial segregation of identified variants was not reported by some investigators.

## Conclusion

The extent to which HS pathogenesis and risk are driven by the shared overlap between comorbid clinical risk factors, such as obesity and smoking, and one’s genetic predisposition remains unknown. The degree to which different variants contribute to the two main pathophysiological processes at the PSU namely hyperkeratosis and inflammation (Nomura, 2020) also remains unelucidated. The weak genotype-phenotype associations observed in HS are similar to other complex diseases. Potentially, this can be attributed to diagnostic delays, phenotypic heterogeneity and pleiotropic genetic effects acting against background modifiers such as changes in the composition of the skin microbiome.

Notwithstanding, the limited number of studies investigating HS by whole exome sequencing, and to a lesser extent by whole genome sequencing, show promising results and highlight the need for patients at the extreme ends of the HS phenotypic spectrum to be identified and prioritized for rare variant screening. To this end, phenotypic evaluation and categorization criteria need to be standardized to facilitate their use and interpretation in clinical care settings (Daxhelet et al., 2020; Frew et al., 2021).

The incompletely understood genomic risk factors of HS warrants further study, possibly by alternative approaches such as genome-wide association studies (GWAS). GWAS should elucidate the contribution of common genomic variants to HS and potentially identify new loci associated with this trait. Such endeavors require large-scale multicenter, collaborative genomic efforts (Daxhelet et al., 2021; Jabbour et al., 2021) which will allow for endotyping (González-Manso et al., 2020), deep phenotyping and, ultimately precision medicine (Robinson, 2012; Delude, 2015) for HS patients. Genetic variables also have the propensity to act as diagnostic and predictive HS biomarkers (Der Sarkissian et al., 2022). The evidence outlined in this review suggests that considering HS as a single disease may be misleading. Conversely, considering a precision-medicine approach tailored to every individual may be unrealistic in health care systems burdened by limited access to genetic testing and escalating health care costs. Important lessons can be derived from studies of other complex traits. Several investigators have shown that precision phenotyping of polygenic disease based on disease mechanisms is superior to traditional clinical classifications as it better identifies patients at risk of complications and can guide therapeutic choices (Ahlqvist et al., 2018). Whether or not dissecting the phenotypic heterogeneity of HS improves clinical outcomes remains to be assessed.
