# Supplementary material for: The Genomic Architecture of Hidradenitis Suppurativa—A Systematic Review
Source: Front Genet. 2022 Mar 23;13:861241. doi: 10.3389/fgene.2022.861241 (PMC8986338; doi:10.3389/fgene.2022.861241)
Supplement: Supplementary file 1 [file DataSheet1.docx]

**Supplementary table 1** Impact of missenses variants associated with hidradenitis suppurativa.

| **Variant** | **Structural Features of mutation site** | **Missense3D** | **Structure – based Stability Predictions** |
| --- | --- | --- | --- |
| ***NCSTN* p.Gly33Arg** | The variant substitutes a hydrophobic neutral residue with a larger positively charged amino acid. The variant is located within the signal peptide. The wild-type glycine is very flexible and the substitution can alter conformation of the N-terminal domain. | Not structurally damaging * | ΔΔG mCSM: Stabilizing (0.0 kcal/mol)  ΔΔG SDM: Stabilizing (0.0 kcal/mol)  ΔΔG DUET: Stabilizing (0.0 kcal/mol)  ΔΔS_Vib_ ENCoM: -0.185 kcal.mol^-1^.K^-1^ (Decrease of molecule flexibility) |
| ***NCSTN* p.Gly61Val** | The substitution replaces a buried glycine with a larger, hydrophobic residue and may impact on the flexibility provided by wild type glycine. The wild type residue is not fully conserved. | The substitution is structurally damaging. It triggers a disallowed phi/psi alert. The phi/psi angles are in favoured region for wild-type residue but outlier region for mutation residue. The substitution also replaces a buried GLY residue (RSA2.3%) with a buried VAL residue (RSA2.8%) | ΔΔG mCSM: Destabilizing (-0.353kcal/mol)  ΔΔG SDM: Destabilizing (-.440 kcal/mol)  ΔΔG DUET: Destabilizing (-0.037 kcal/mol)  ΔΔS_Vib_ ENCoM: -0.781 kcal.mol^-1^.K^-1^ (Decrease of molecule flexibility) |
| ***NCSTN* p.Val75Ile** | Substitution replaces an exposed hydrophobic VAL with larger buried hydrophobic ILE residue. Residue not fully conserved. | Not structurally damaging | ΔΔG mCSM: Destabilizing (-0.665kcal/mol)  ΔΔG SDM: Destabilizing (-0.830 kcal/mol)  ΔΔG DUET: Destabilizing (-0.478 kcal/mol)  ΔΔS_Vib_ ENCoM: -0.225 kcal.mol^-1^.K^1^ (Decrease of molecule flexibility) |
| ***NCSTN* p.Asp185Asn** | The wild-type residue ASP is exposed negatively charged with RSA 29.4% and the variant residue ASN is exposed uncharged with RSA 20.3%. The position is not fully conserved.  The variant substitutes a hydrophobic neutral Pro residue with a larger, positively charged Arg residue. The wild type Pro is conformationally rigid. | Not structurally damaging | ΔΔG mCSM: Destabilizing (-0.399kcal/mol)  ΔΔG SDM: Stabilizing (0.250 kcal/mol)  ΔΔG DUET: Destabilizing (-0.182 kcal/mol)  ΔΔS_Vib_ ENCoM: -0.164 kcal.mol^-1^.K^-1^ (Decrease of molecule flexibility) |
| ***NCSTN* p.Pro211Arg** | The variant residue is bigger than the wild-type residue. The variant residue introduces a positive charge. The wildtype residue is more hydrophobic than the variant residue. The wild-type residue, a proline, is known to be very rigid and therefore induce a special backbone conformation which might be required at this position. The mutation can disturb this special conformation. | Not structurally damaging | ΔΔG mCSM: Destabilizing (-0.652kcal/mol)  ΔΔG SDM: Stabilizing (1.620 kcal/mol)  ΔΔG DUET: Destabilizing (-0.036 kcal/mol  ΔΔS_Vib_ ENCoM: -0.188 kcal.mol^-1^.K^-1^ (Decrease of molecule flexibility) |
| **NCSTN p.Gln216Pro** | The variant residue is smaller than the wild type residue. Them variant residue is more hydrophobic than the wild-type residue. This can result in loss of hydrogen bonds and/or disturb correct folding. The wild type residue is predicted to be located in an a-helix. Proline disrupts an a-helix when not located at one of the first 3 positions of that helix. In case of the variant at hand the helix will be disturbed and this can have effets on the structure of the protein. | This substitution triggers disallowed phi/psi alert. The phi/psi angles are in favored region for wild-type residue but outlier region for variant residue* | ΔΔG mCSM: -0.388 kcal/mol (Destabilizing)  ΔΔG SDM: -1.810 kcal/mol (Destabilizing)  ΔΔG DUET: -0.637 kcal/mol (Destabilizing)  ΔΔS_Vib_ ENCoM: -0.021 kcal.mol^-1^.K^-1^ (Decrease of molecule flexibility) |
| ***NCSTN* p.Glu296Gly** | The substitution replaces a negatively charged residue with a smaller, more flexible, hydrophobic, neutral residue | The substitution is structurally damaging. It replaces a buried charged residue (Glu, RSA 0.0%) with an uncharged residue (Gly). It also leads to the expansion of the cavity volume by 139.9 Å^3^ | ΔΔG mCSM: Destabilizing (-0.380kcal/mol)  ΔΔG SDM: Destabilizing (-1.000 kcal/mol)  ΔΔG DUET: Destabilizing (-0.523 kcal/mol  ΔΔS_Vib_ ENCoM: 1.029 kcal.mol^-1^.K^-1^ (Increase of molecule flexibility) |
| ***NCSTN* p.Ala315Val** | The variant residue is bigger than the wild-type residue The wild type residue is buried in the core of the protein. The larger, variant residue probably does not fit in the protein core. | Not structurally damaging | ΔΔG mCSM: Destabilizing (-0.037 kcal/mol)  ΔΔG SDM: Destabilizing (-1.270 kcal/mol)  ΔΔG DUET: Destabilizing (-0.067 kcal/mol)  ΔΔS_Vib_ ENCoM: -0.227 kcal.mol^-1^.K^-1^ (Decrease of molecule flexibility) |
| ***NCSTN* p.Ala410Val** | The variant residue is bigger than the wild-type residue | Not structurally damaging | ΔΔG mCSM: Destabilizing (-0.432 kcal/mol)  ΔΔG SDM: Destabilizing (-0.210 kcal/mol)  ΔΔG DUET: Destabilizing (-0.269 kcal/mol)  ΔΔS_Vib_ ENCoM: -0.455 kcal.mol^-1^.K^-1^ (Decrease of molecule flexibility) |
| ***NCSTN* p.Gly576Val** | The variant residue is bigger, more flexible and more hydrophobic than the wild type residue | The substitution is structurally damaging as it replaces a glycine located in a bend curvature. | ΔΔG mCSM: Destabilizing (-0.625kcal/mol)  ΔΔG SDM: Destabilizing (-0.970 kcal/mol)  ΔΔG DUET: Destabilizing (-0.628 kcal/mol)  ΔΔS_Vib_ ENCoM: -1.420 kcal.mol^-1^.K^-1^ (Decrease of molecule flexibility) |
|  |  |  |  |
| ***PSEN1* p.Glu318Gly** | The variant residue is smaller, more hydrophobic and more flexible than the wild type Glu. The substitution alters charge at this residue/ | Not structurally damaging* | ΔΔG mCSM: Destabilizing (-0.014kcal/mol)  ΔΔG SDM: Stabilizing (0.510 kcal/mol)  ΔΔG DUET: Stabilizing (0.328 kcal/mol)  ΔΔSVib ENCoM: 0.614 kcal.mol-1.K-1 (Increase of molecule flexibility) |
|  | | | |
| ***PSENEN* p.Leu65Arg** | The variant residue is larger than the wild type Leu. Since the variant lies in a transmembrane domain, the size difference may also disrupt the contacts within the lipid-membrane. The variant substitutes a neutral hydrophobic amino acid with a positively charged residue. | Not structurally damaging | ΔΔG mCSM: Destabilizing (-0.286 kcal/mol)  ΔΔG SDM: Stabilizing (0.090 kcal/mol)  ΔΔG DUET: Stabilizing (0.024 kcal/mol)  ΔΔS_Vib_ ENCoM: 0.237 kcal.mol^-1^.K^-1^ (Increase of molecule flexibility) |
|  | | | |
| ***APH*1 p.His170Arg** | The variant residue is larger than the wild type His. The size difference can affect contacts with the lipid membrane. The variant introduces a charge, this can cause repulsion of ligands or other residues with the same charge. | The substitution disrupts all side-chain / side-chain H-bond(s) and/or side-chain / main chin bond(s) H-bonds formed by a buried His residue (RSA 0.0%) *   \| Donor \| Acceptor \| Distance \| Type \| \| --- \| --- \| --- \| --- \| \| WILD TYPE \| \| \| \| \| A0087-ARG Nh1 \| A0170-HIS ND1 \| 3.12 \| SS \| \| A0128-Ser OG \| A0170-HIS NE2 \| 3.33 \| SS \| \| Mut \| \| \| \| \| No hydrogen bond found \| \| \| \| | ΔΔG mCSM: -1.302 kcal/mol (Destabilizing)  ΔΔG SDM: -1.370 kcal/mol (Destabilizing)  ΔΔG DUET: -1.288 kcal/mol (Destabilizing)  ΔΔS_Vib_ ENCoM: -0.153 kcal.mol^-1^.K^-1^ (Decrease of molecule flexibility) |
|  | | | |
| ***PSTPIP1* p.Glu250Gln** | The variant residue is smaller than the wild type residue. The wild type residue negatively charged whilst the mutation residue is neutral. The variant residue is more hydrophobic than the wild type residue. This can result in loss of hydrogen bonds and/or disturb correct folding. The mutation is located within the F-BAR domain of the protein. The mutation introduces Glycine, which has different properties from WT Glutamate. This can disrupt this domain and abolish the protein’s functions. The introduced Glycine residue is very flexible and can disrupt the rigidity of the protein at this position.  Only Glutamate is found in this position. Mutation of a fully conserved residue is usually damaging for the protein, especially since the variant and wild type residue are dissimilar. | Not structurally damaging* | ΔΔG mCSM: -0.661 kcal/mol (Destabilizing)  ΔΔG SDM: -1.140 kcal/mol (Destabilizing)  ΔΔG DUET: -0.572 kcal/mol (Destabilizing)  ΔΔS_Vib_ ENCoM: -0.010 kcal.mol^-1^.K^-1^ (Decrease of molecule flexibility) |
| ***PSTPIP1 p.Thr255Met*** | The variant residue is larger and is more hydrophobic than the wild type residue. This can result in loss of hydrogen bonds and/or disrupt correct folding. The mutation is located within the F-BAR domain. The mutation introduces Threonine, which has different properties from wild type methionine. This can disrupt this domain and abolish the protein’s functions. | Not structurally damaging* | ΔΔG mCSM: 0.096 kcal/mol (Stabilizing)  ΔΔG SDM: 1.300 kcal/mol (Stabilizing)  ΔΔG DUET: 0.597 kcal/mol (Stabilizing)  ΔΔS_Vib_ ENCoM: 0.074 kcal.mol^-1^.K^-1^ (Increase of molecule flexibility) |
| ***PSTPIP1* p.Glu277Asp** | The variant residue is smaller than the wild type residue. The position is not conserved. | Not structurally damaging* | ΔΔG mCSM: Destabilizing (-0.372 kcal/mol)  ΔΔG SDM: Destabilizing (-0.520kcal/mol)  ΔΔG DUET: Destabilizing (-0.157 kcal/mol)  ΔΔSVib ENCoM: -0.173 kcal.mol-1.K-1 (Decrease of molecule flexibility) |
| ***PSTPIP1* p.Tyr345Cys** | The variant residue is smaller and more hydrophobic than the wild-type Tyr.  The wild type residue is predicted (using Reprof software) to be in its preferred secondary structure, a β-strand. The variant prefers to be in another secondary structure, therefore the local conformation will be slightly destabilized. Mutagenesis experiments at this position have shown that mutation of the Tyr residue at his position can lead to decrease binding to MEFV. | Not structurally damaging* | ΔΔG mCSM: Stabilizing (0.006 kcal/mol)  ΔΔG SDM: Stabilizing (0.130kcal/mol)  ΔΔG DUET: Stabilizing (0.193 kcal/mol)  ΔΔSVib ENCoM: 3.680 kcal.mol-1.K-1 (Increase of molecule flexibility) |
| ***PSTPIP1* p.Arg405Cys** | The variant residue is smaller, uncharged and hydrophobic. The variant lies within an SH3 domain and introduces an amino acid with different properties and may result in domain disruption potentially abolishing its function. | No structural damage detected | ΔΔG mCSM: Destabilizing (-0.532 kcal/mol)  ΔΔG SDM: Stabilizing (-0.009kcal/mol)  ΔΔG DUET: Destabilizing (-0.358 kcal/mol)  ΔΔSVib ENCoM: 0.459 kcal.mol-1.K-1 (Increase of molecule flexibility) |
| **NLRP3 p.Gln703Lys** | The variant residue is bigger than the wild type residue. The variant residue introduces a positive charge. | No structural damage detected* | ΔΔG mCSM: 0.011 kcal/mol (Stabilizing)  ΔΔG SDM: 0.340 kcal/mol (Stabilizing)  ΔΔG DUET: 0.324 kcal/mol (Stabilizing)  ΔΔS_Vib_ ENCoM: -4.936 kcal.mol^-1^.K^-1^ (Decrease of molecule flexibility) |
| **IL1RN p.Ala124Thr** | The wildtype residue is small and more hydrophobic than the variant residue. | No structural damage detected* | ΔΔG mCSM: -0.823 kcal/mol (Destabilizing)  ΔΔG SDM: -0.990 kcal/mol (Destabilizing)  ΔΔG DUET: -0.565 kcal/mol (Destabilizing)  ΔΔS_Vib_ ENCoM: -0.207 kcal.mol^-1^.K^-1^ (Decrease of molecule flexibility) |
| **NOD2 p.Arg675Trp** | The variant residue is bigger than the wild type residue. The wild type Arg residue is positively charged whilst the variant residue is neutral. The variant residue is more hydrophobic than the wild type residue. This can result in loss of hydrogen bonds and/or disturb correct folding. | No structural damage detected* | ΔΔG mCSM: -0.174 kcal/mol (Destabilizing)  ΔΔG SDM: 0.100 kcal/mol (Stabilizing)  ΔΔG DUET: -0.294 kcal/mol (Destabilizing)  ΔΔS_Vib_ ENCoM: -0.340 kcal.mol^-1^.K^-1^ (Decrease of molecule flexibility) |
| **NOD2 p.Gly908Arg** | The variant residue is bigger than the wild type residue. The variant residue introduces a positive charge, as the wild type residue is neutral.  Only Glycine residue is found at position 908. The mutation and the WT residue are not very similar. Based on this conservation the mutation is probably damaging to the protein.  The mutated residue is located in a domain that is important for binding of other molecules. Mutation of the residue might disturb this function.  The torsion angles for this residue are unusual. Only wild type GLY is flexible enough to make the torsion angles. The mutation forces the local backbone into an incorrect conformation and will disturb the local structure. | No structural damage detected* | ΔΔG mCSM: -0.744 kcal/mol (Destabilizing)  ΔΔG SDM: -1.380 kcal/mol (Destabilizing)  ΔΔG DUET: -0.826 kcal/mol (Destabilizing)  ΔΔS_Vib_ ENCoM: -2.082 kcal.mol^-1^.K^-1^ (Decrease of molecule flexibility) |
| **NOD2 p.Leu975Val** | The variant residue is smaller than the wild type residue. The mutation lies within a stretch of residues that is repeated in the protein (LRR 7). The variant might disturb this repeat and consequently any function this repeat may have. The mutation also lies in a domain that is important for binding of other molecules, and can have implication on function. | No structural damage detected* | ΔΔG mCSM: -1.712 kcal/mol (Destabilizing)  ΔΔG SDM: -3.210 kcal/mol (Destabilizing)  ΔΔG DUET: -2.209 kcal/mol (Destabilizing)  ΔΔS_Vib_ ENCoM: 0.363 kcal.mol^-1^.K^-1^ (Increase of molecule flexibility) |

***No experimentally derived structure of this region available, prediction was performed using predicted structure from AlphaFold. Variant lies in a region of the protein predicted with low/very low confidence. IL1RN, NOD2, NL3 structural models are based on the canonical sequence**

**Supplementary Table 2** Variants associated with Syndromic HS.

|  | **Gene** | **Variant** | **Protein Change** |
| --- | --- | --- | --- |
| **PASH Syndrome** *^▲^* | **NCSTN** | c.1635C>G | p.Tyr545Ter |
|  | **NCSTN** | c.344_351del | p.Thr115AsnfsTer20 |
|  | **PSENEN** | c.228_229insCACC | p.Ile77HisfsTer45 |
|  | **MEFV** | Chr16:3293407T>C  RS61752717 | p.M694V |
|  | **MEFV** | Chr16:3293310A>G  Rs28940579 | p.V726A |
|  | **MEFV** | Chr16:3293880A>G  Rs11466045 | p.I591T |
|  | **NOD2** | c.2023C>T | p.Arg675Trp |
|  | **NOD2** | c.2722G>C | p.Gly908Arg |
|  | **NOD2** | c.2923C>G | p.leu975Val |
|  | **IL1RN** | c.370G>A | p.Ala106Thr |
|  | **PSTPIP1** | c.1213C>T | p.Arg450Cys |
|  | **NLRP3** | c.2107C>A | p.Glyn703Lys |
| **PAPASH Syndrome***•* | **PSTPIP1** | c.-413_-402dupCCTGCCTGCCTG^•^ |  |
|  | **PSTPIP1** | c.748G>C | p.Glu250Gln |
|  | **PSTPIP1** | c.831G>T | p.Glu277Asp |
|  | **MEFV** | c.2082G>A | p.Met694Ile |
| **SAPHO syndrome** *** | **NCSTN** | c.278del | p.Pro93LeufsTer15 |
| **PPHSF syndrome** *^$^* | **PSTPIP1** | c.764C>T | p.Thr255Met |
